# TKI rotation-induced persistent deep molecular response in multi-resistant blast crisis of Ph+ CML

**DOI:** 10.18632/oncotarget.15481

**Published:** 2017-02-18

**Authors:** Peter Valent, Susanne Herndlhofer, Mathias Schneeweiß, Bernd Boidol, Anna Ringler, Stefan Kubicek, Karoline V. Gleixner, Gregor Hoermann, Emir Hadzijusufovic, Leonhard Müllauer, Wolfgang R. Sperr, Giulio Superti-Furga, Christine Mannhalter

**Affiliations:** ^1^ Department of Internal Medicine I, Division of Hematology and Hemostaseology, Medical University of Vienna, Austria; ^2^ Ludwig Boltzmann Cluster Oncology, Medical University of Vienna, Austria; ^3^ CeMM Research Center for Molecular Medicine of the Austrian Academy of Sciences, Austria; ^4^ Department of Pathology, Medical University of Vienna, Austria; ^5^ Department of Laboratory Medicine, Medical University of Vienna, Austria; ^6^ Center for Physiology and Pharmacology, Medical University of Vienna, Austria

**Keywords:** CML, ponatinib, nilotinib, BCR-ABL1 mutations, drug resistance

## Abstract

In chronic myeloid leukemia (CML) resistance against one or more BCR-ABL1 tyrosine kinase inhibitors (TKI) remains a clinical challenge. Preclinical data suggest that TKI combinations may overcome resistance. We report on a heavily pre-treated 78 year-old female patient with CML who developed multi-resistant blast crisis with bone marrow fibrosis and a Ph- clone. Treatment with ponatinib resulted in blast cell clearance, decrease in fibrosis, and disappearance of *BCR-ABL1*, but also in severe thrombocytopenia with bleedings requiring platelet transfusions. We therefore switched from ponatinib to bosutinib. During bosutinib, platelet counts recovered. However, after 6 months, *BCR-ABL1* mRNA levels increased to > 1%. Therefore, we ´switched back´ to ponatinib, and this was again followed by disappearance of *BCR-ABL1* and a decrease in platelets. During the next 2 years, we applied ponatinib and bosutinib in continuous rotation-cycles and added hydroxyurea in order to suppress all sub-clones and to balance between efficacy and potential side effects following the principle of personalized medicine. With this approach the patient remained in complete molecular response and reached normal blood counts and a normal quality of life without vascular or other side effects. In conclusion, TKI rotation is a novel potent approach to suppress multiple resistant sub-clones and to balance between clinical efficacy and side effects in patients with advanced CML. Clinical trials are now warranted to show that TKI-rotation is in general safe and effective in these patients.

## INTRODUCTION

Chronic myeloid leukemia (CML) is a hematopoietic neoplasm characterized by expansion and accumulation of myeloid progenitor cells exhibiting the Philadelphia (Ph) chromosome and the related oncoprotein, BCR-ABL1 [1–3]. Usually, patients are diagnosed in the chronic phase (CP) of the disease where most leukemic cells are addicted to the tyrosine kinase (TK) activity of BCR-ABL1. As a consequence the BCR-ABL1-targeting drug imatinib can produce complete and durable cytogenetic responses as well as major or even complete molecular responses (MR3-MR5) in a majority of ´prescription-adherent´ CP patients [4–7]. However, not all patients (even when ´adherent´) are long-term responders. Rather, in about 20–30% of the patients, acquired resistance against imatinib is found [7–10]. In these patients, imatinib-resistant mutants of BCR-ABL1 are detected frequently. For these cases, second-generation BCR-ABL1 TK inhibitors (TKI) are available and are usually prescribed to control the disease [11–15]. These TKI include nilotinib, dasatinib, and bosutinib. All three agents received approval for treatment of imatinib-resistant or -intolerant CML. However, not all mutant forms of BCR-ABL1 are recognized by these drugs. A special problem is the T315I mutation of *BCR-ABL1* that is well-known to confer resistance against imatinib, nilotinib, dasatinib, and bosutinib [16–18]. For these patients, stem cell transplantation (SCT) has to be considered. In addition, these patients often respond to ponatinib, a novel TKI that blocks most mutant forms of BCR-ABL1, including T315I [19–22]. Indeed, it has been shown that ponatinib can induce major or even complete cytogenetic responses in patients with TKI-resistant CML in whom neoplastic cells display BCR-ABL1 T315I [20–22]. However, unfortunately, the administration of ponatinib is associated with clinically relevant side effects, including thrombocytopenia, an increase in pancreatic enzymes (rarely pancreatitis), and vascular occlusive events [20–23]. Of special concern is the occurrence of vascular occlusive diseases, as these events may lead to irreversible organ damage or even death [20–23]. Therefore, current attempts focus on the management and avoidance of such adverse events in patients treated with ponatinib. One strategy may be to decrease the dose of ponatinib from 45 mg daily to 30 or even 15 mg daily. However, it remains uncertain whether efficacy is comparable when lower doses of the drug are applied, especially in advanced CML. Another strategy may be to administer co-medication that can counteract atherosclerosis and thrombosis [23]. However, such agents, like aspirin or statins, may also produce side effects and may be problematic in ponatinib-treated patients, especially when thrombocytopenia occurs.

We here describe a patient who received ponatinib because of TKI-resistant blast crisis (BC) of CML accompanied by bone marrow (BM) fibrosis and a Ph-negative sub-clone. In this patient, ponatinib was effective in eradicating the dominant CML clone, but also induced severe thrombocytopenia. A switch to bosutinib resulted in improved platelet counts, but was also followed by a molecular relapse. Subsequently, we applied ponatinib and bosutinib in rotation and added hydroxyurea (HU), with the aim to suppress all sub-clones, to avoid side effects, and to preserve efficacy at the same time, thus following the principles of personalized medicine.

## RESULTS

### Characterization of the disease at the time of blast crisis (CR)

At the time of BC, the BM showed marked fibrosis as well as a huge increase in myeloblasts expressing KIT, CD33 and other myeloid antigens. Although the patient had also developed BM fibrosis and a Ph-negative subclone, most aspirable leukemic cells and circulating blast cells displayed *BCR-ABL1*. We also examined the peripheral blood (PB) and BM for the presence of mutations in *JAK2*, *CALR*, *MPL*, *KIT*, and various AML-related fusion genes. However, no mutations in these genes were detected in the patients´ blast cells in our analyses. Blast cells were tested positive for the *BCR-ABL1* mutations L248V and K274del, whereas the F317L mutation that had been detected earlier in our patient, was not found by Sanger sequencing, suggesting that this sub-clone and its neoplastic stem cells had been eliminated or were controlled by TKI therapy.

### Initial response to ponatinib

**Figure 1:**
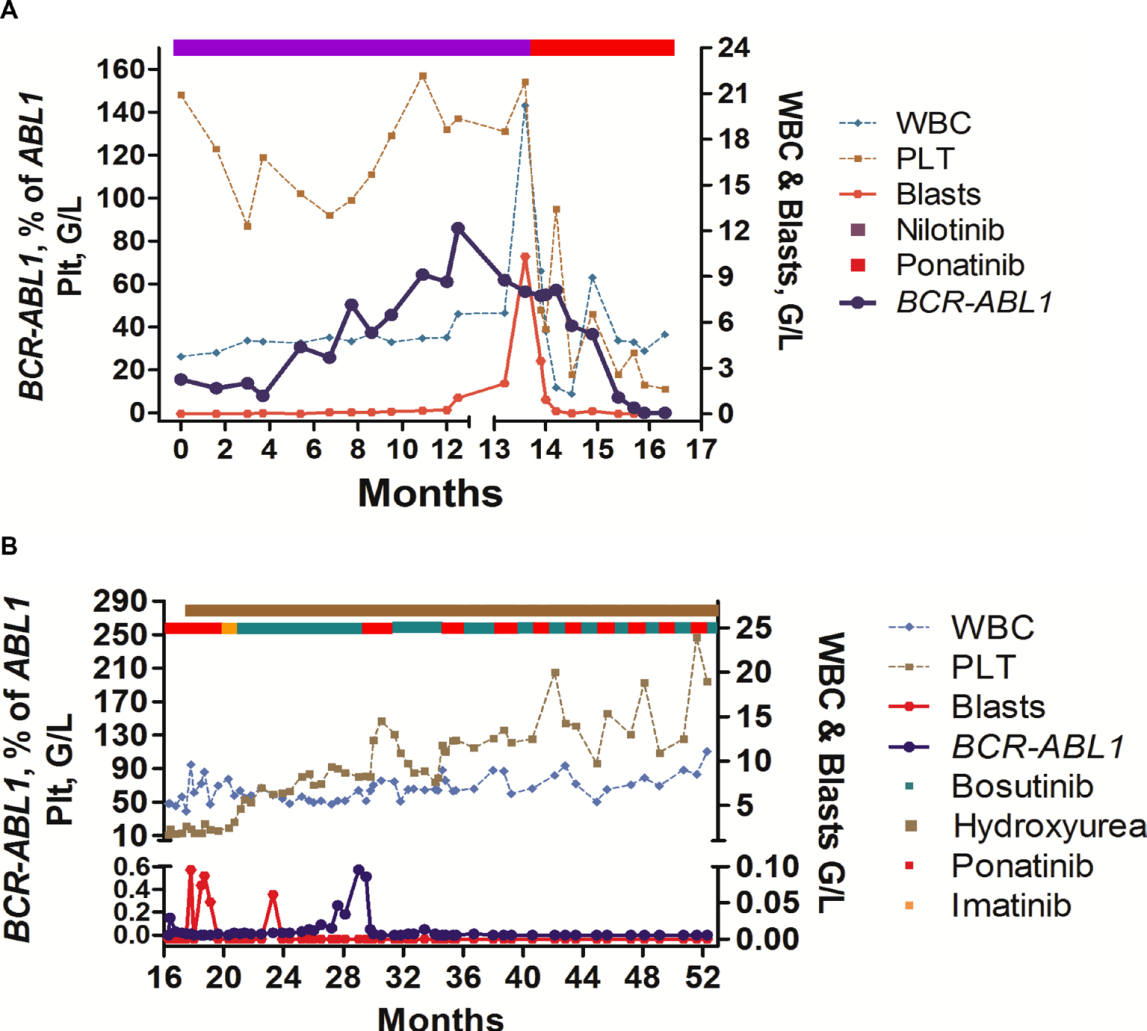
Overview of clinical course and response to TKI rotation therapy (**A**) Response to treatment with nilotinib and ponatinib, and influence of TKI therapy on blood counts and *BCR-ABL1* mRNA levels: The patient was treated with nilotinib after resistance against dasatinib had been documented. During nilotinib, *BCR-ABL1* decreased but did not disappear. After several months, *BCR-ABL1* increased again and cytopenia developed. Finally, the patient developed blast crisis and treatment with ponatinib was initiated. In response to ponatinib, *BCR-ABL1* mRNA levels decreased, but the patient developed severe thrombocytopenia. (**B**) Response to treatment with ponatinib/bosutinib-rotation and hydroxyurea (HU): Because of thrombocytopenia, the patient was switched to bosutinib (after a short phase of imatinib-bridging). During bosutinib, platelet counts recovered slowly, but *BCR-ABL1* increased again. Therefore, we switched back to ponatinib, and finally decided to apply ponatinib and bosutinib in continuous rotation-cycles together with low-dose HU. Under this therapy, blood counts normalized, the patient entered a stable continuous complete response, and has a normal quality of life without major side effects.

After an initial start-dose of 45 mg ponatinib per day (first week) the patient received 30 mg ponatinib daily. Within a few weeks, blast cells decreased substantially and disappeared in the PB. In addition, *BCR-ABL1* mRNA levels decreased significantly. However, the patient also developed severe thrombocytopenia and bleedings requiring platelet transfusions as well as marked neutropenia (Table 1, Figure 1). We therefore decided to discontinue ponatinib. The patient was shortly kept on imatinib and then switched to bosutinib (300 mg/day) on a compassionate use program. During bosutinib, platelet counts recovered, and the clinical situation of the patient improved. However, after a few months, *BCR-ABL1* increased again and we decided to start rotation therapy employing ponatinib and bosutinib (Figure 1). In addition, the patient received HU to keep the Ph-negative sub-clone under control.

**Table 1:** Major clinical variables before and during TKI-rotation therapy

PO-BOmonths*	WBC G/L	PLTG/L	% blasts		BMFibrosis	LDHU/L	BCR-ABL1% in PB cells**	% Ph+ cellsin BM samples
			PB	BM				
−18	04.82	235	00	nd	yes	292	26.463	80
−15	03.65	222	00	nd	nd	374	28.283	nd
−12	04.01	123	00	nd	nd	321	11.548	nd
−09	04.29	101	01	nd	nd	339	29.549	nd
−06	04.77	099	02	nd	nd	382	52.293	nd
−03	05.12	129	02	nd	nd	504	64.341	nd
−01	08.11	154	23	32	nd	714	86.000	100
00	24.41	088	36	nd	nd	1,033	56.480	nd
+01	08.93	046	02	nd	nd	552	36.573	nd
+03	04.93	008	00	nd	nd	472	0.026	nd
+06	05.49	027	00	nd	nd	579	0.011	nd
+09	06.18	059	01	nd	nd	338	0.023	nd
+12	05.29	070	00	nd	nd	316	0.036	nd
+15	06.68	080	00	nd	nd	230	0.573	nd
+18	07.67	131	00	< 5	no	230	< 0.0032	nd
+21	07.80	110	00	nd	nd	206	< 0.0032	nd
+24	08.95	126	00	nd	nd	215	< 0.0032	nd
+27	10.99	164	00	nd	nd	242	< 0.0032	nd
+30	10.09	175	00	nd	nd	263	< 0.0032	nd
+33	07.30	131	00	nd	nd	231	< 0.0032	nd
+36	07.39	189	00	nd	nd	278	< 0.0032	nd

Abbreviations: PO, ponatinib; BO, bosutinib; PLT, platelet count; PB, peripheral blood; BM, bone marrow; LDH, lactate dehydrogenase; nd, not done.

*Months before or after the start of ponatinib-bosutinib rotation therapy.

**BCR-ABL1 mRNA levels were determined by qPCR and expressed as percent of *ABL1* after adjusting to the international scale (IS).

### Clinical response to rotation therapy employing ponatinib and bosutinib + HU

During treatment with ponatinib and bosutinib in rotation-cycles combined with low dose HU (1 g/day), *BCR-ABL1* decreased to MR4.5 and finally to undetectable levels (Figure 1). In addition, blood counts normalized and a reinvestigation of the BM revealed a marked decrease in BM fibrosis. After a total observation-time of 55 months, the patient is in continuous MR4.5 with normal or near normal blood counts and no signs of a relapse with a Ph+ or a Ph-negative disease. Because of the potential risk of side effects, because of concomitant HU therapy, and because of continuous BCR-ABL1-negativity, both drugs were maintained at relatively low doses (bosutinib: 300 mg/day; and ponatinib at 30 mg/day).

### Safety assessment and management of side effects

rotation therapy was well tolerated without major side effects. Rather the patient reported on an increase in her quality of life. The only complaint was a recurrent mild pleural effusion that had been already documented during treatment with dasatinib, and was again detectable (and slightly increased) during treatment with bosutinib. Therefore, the patient also received low dose prednisolone (25 mg/day for 3 days followed by 12.5 mg/day for another week) during therapy with bosutinib in each cycle. In addition, the patient was maintained on aspirin as prophylactic treatment to minimize the risk of ponatinib-induced thrombosis. Based on the well-known side effects occurring in patients treated with nilotinib and ponatinib, we also examined vascular and metabolic parameters before and during treatment with ponatinib. However, no vascular event and no substantial metabolic changes were noted during rotation. In addition, all serum parameters, including pancreatic enzymes, HbA1c and cholesterol levels, remained within normal range. The European Society of Cardiology (ESC) score was 2 (indicating an intermediate risk to develop a cardiovascular event) before and during treatment with ponatinib/bosutinib.

### Histologic examinations before and after therapy with TKI rotation

BM histology-results were obtained and compared before and during treatment with TKI rotation therapy. Prior to rotation therapy, a marked BM fibrosis was seen. In addition, the BM revealed signs of dysplasia and myeloproliferation. At the time of BC no histology was obtained. After successful therapy with TKI rotation, the BM showed an almost complete resolution of fibrosis and a normal blast cell (CD34+ cell) count.

### Molecular studies and *HUMARA* pattern

**Figure 2:**
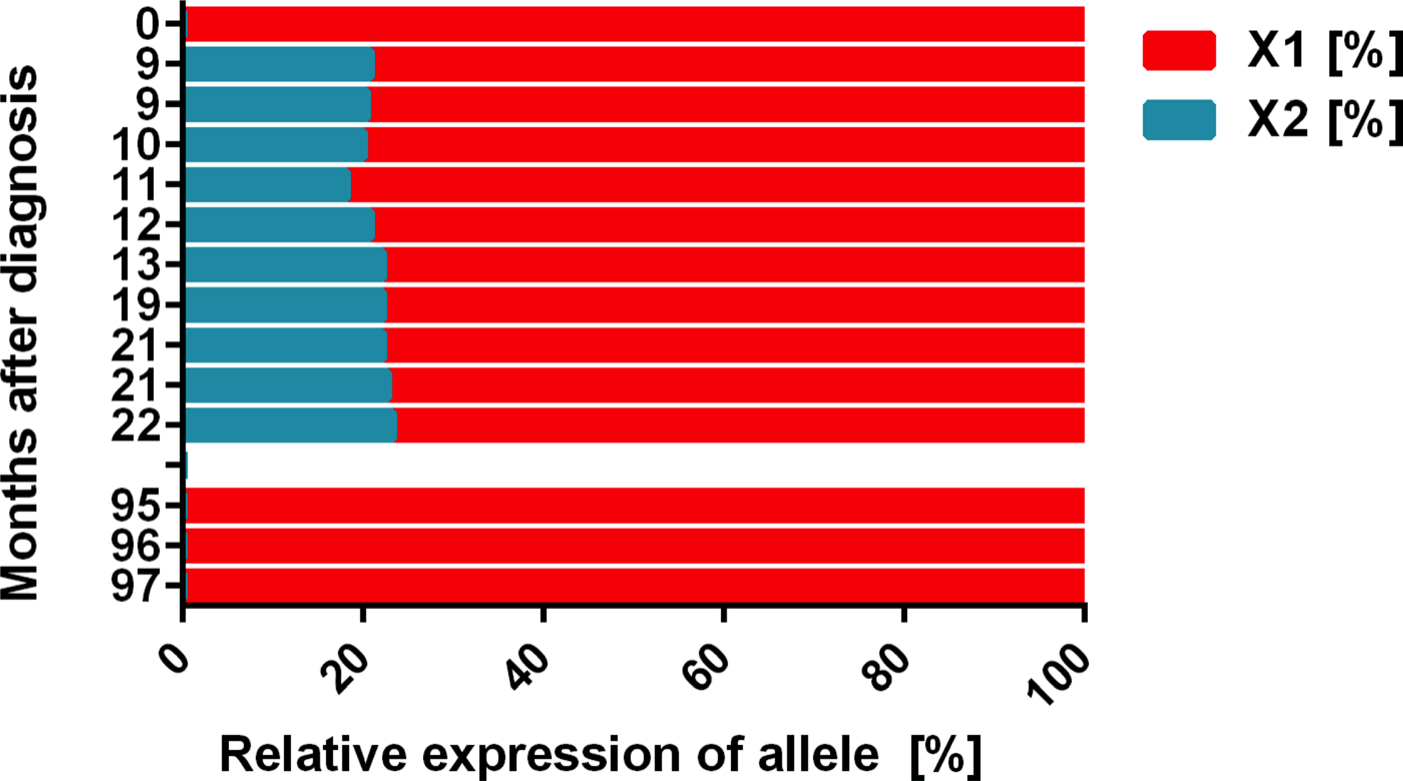
Clonal evolution during TKI therapy as assessed by HUMARA The HUMARA assay was performed with peripheral blood mononuclear cells in certain time intervals (from 2005 = month 0) as indicated. As assessed by HUMARA, a monoclonal pattern was seen in 2005 (month 0) and at the time of relapse with a Ph-negative clone when bone marrow (BM) fibrosis was detected (months 95–97). However, after successful treatment with dasatinib and nilotinib (months 9–22), a polyclonal pattern was obtained by HUMARA testing.

Based on the detection of a Ph-negative subclone, we determined the clonality status before and during treatment with BCR-ABL1 TKI. Before starting TKI therapy, a monoclonal pattern was detected by the HUMARA assay. During successful treatment with dasatinib and ponatinib, a polyclonal pattern was seen (Figure 2). However, at the time of occurrence of BM fibrosis, the polyclonal pattern was lost and a monoclonal pattern was observed. At that time, *BCR-ABL1* levels were still below 1%. These data suggest that the Ph-negative (MPN/MDS-like) sub-clone that produced the massive BM fibrosis was monoclonal in nature even though no *BCR-ABL1* was expressed.

### Drug combination effects *in vitro*

**Figure 3:**
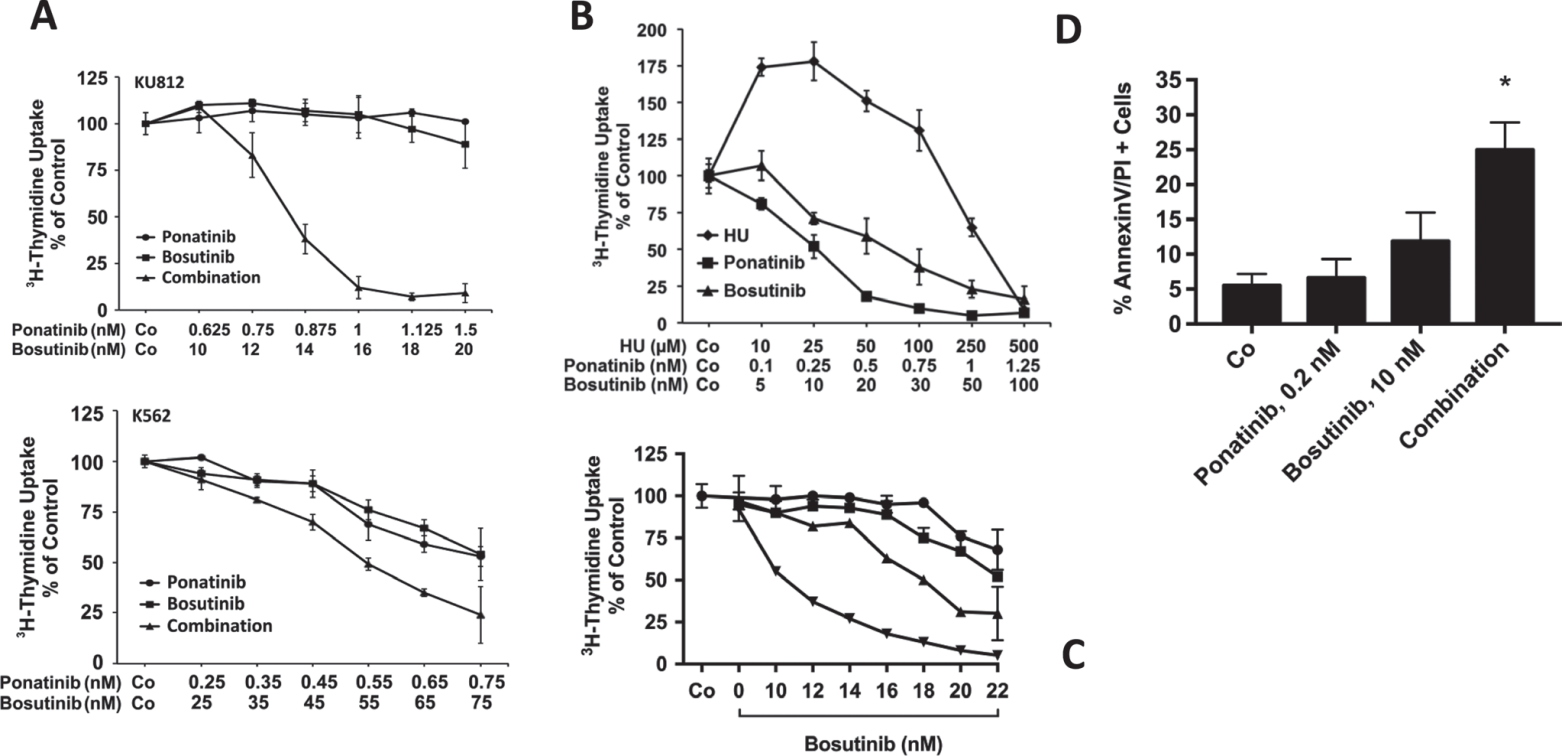
Drug combination effects in CML cells (**A**) KU812 cells (upper panel) and K562 cells (lower panel) were incubated in control medium (Co), with various concentrations of ponatinib or bosutinib, or with a combination of both drugs at a fixed ratio (KU812 at 1:16; K562 at 1:100) at 37°C for 48 hours. Thereafter, ^3^H-thymidine was added for 16 hours, and uptake of ^3^H-thymidine was measured in a β-counter. Results are expressed as percent of medium control and represent the mean ± S.D. of quadruplicates. In case of KU812 cells, the drug combination was found to be highly synergistic, whereas in K562 cells, mostly additive effects were obtained. (**B**) Primary patient-derived blast cells were incubated in control medium (Co) or with increasing concentrations of hydroxyurea (HU), ponatinib, or bosutinib at 37°C and 5% CO_2_ for 48 hours as indicated. Then, ^3^H-thymidine uptake was measured. Results are expressed as percent of control and represent the mean ± S.D. of triplicates. (**C**) KU812 cells were incubated with ponatinib (0.1 nM: ■-■, 0.5 nM: ▲−▲, 1.0 nM: ▼-▼, or control medium: •-•) at 37°C for 4 hours. Then, cells were washed and incubated in control medium (Co) or bosutinib at various concentrations as indicated for another 48 hours. Thereafter, ^3^H-thymidine uptake was measured. Results are expressed as percent of control and represent the mean ± S.D. of triplicates. (**D**) KU812 cells were incubated in control medium (Co), ponatinib (0.2 nM), bosutinib (10 nM), or a combination of both drugs at 37°C for 48 hours. Thereafter, the percentage of AnnexinV/PI-positive cells was determined by flow cytometry. Results represent the mean ± S.D. of 3 independent experiments. Asterisk (*): *p* < 0.05 compared to control.

**Figure 4:**
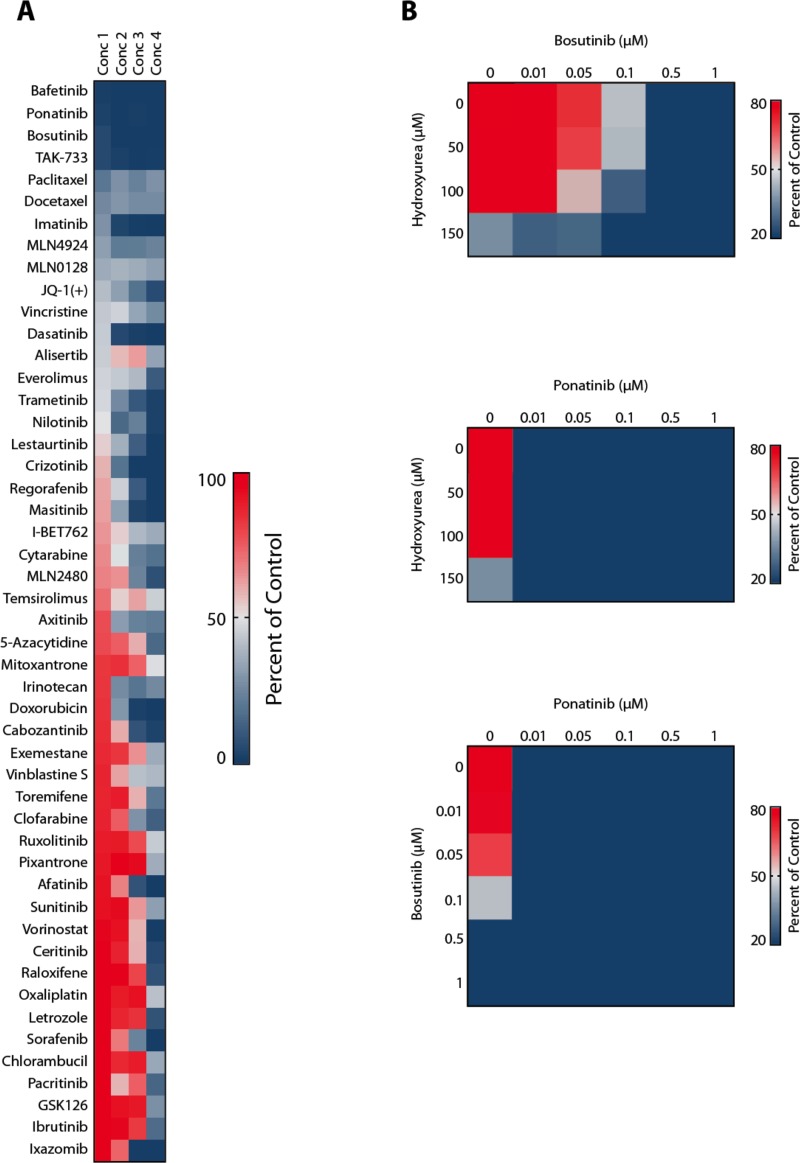
High capacity drug testing using patient-derived CML blast crisis cells (**A**) Nanoliter amounts of BCR-ABL1 TKI and other drugs were pre-seeded at 4 defined concentrations into 384-well microtiter plates using acoustic compound transfer. CML cells (10,000 per well) were automatically plated and incubated with drugs at 37°C for 72 hours. Thereafter, cell viability was determined using Cell-Titer Glo assay. Responses were determined by measuring the percentage of viable cell (relative to control) at the 4 drug concentrations applied. (**B**) Cells were exposed to the drug combinations ´bosutinib+hydroxyurea´ (upper panel), ´ponatinib+hydroxyurea´ (middle panel), and ´ponatinib+bosutinib´ (lower panel) in the robotic drug testing assay.

In order to confirm drug effects on CML cells, we performed *in vitro* studies using two Ph^+^ CML cell lines as well as ponatinib and bosutinib. In these experiments, we were able to show that the two TKI produce strong cooperative anti-neoplastic effects on proliferation in KU812 cells and K562 cells (Figure 3A). In addition, we were able to show that ponatinib and bosutinib inhibit the proliferation of primary patient-derived blast cells in our *in vitro* experiments (Figure 3B). In a next step, we applied ponatinib (4 hours) and bosutinib (48 hours) sequentially to KU812 cells to mimic *in vivo* drug exposure conditions, and measured cell proliferation. As visible in Figure 3C, clear anti-neoplastic cooperative drug effects were also obtained in these experiments. Furthermore, we were able to show that ponatinib and bosutinib produce cooperative apoptosis-inducing effects in KU812 cells (Figure 3D). Finally, the superior anti-neoplastic effects of bosutinib and ponatinib on patient-derived leukemic cells were confirmed in a high-throughput viability assay (Figure 4A). In this assay the effects of bosutinib and ponatinib on viability were stronger compared to the effects of nilotinib, the agent under which the TKI-resistant sub-clones had emerged (Figure 4A). We also applied drug combinations in this assay. In particular, both TKI were combined with each other and with HU, the third inhibitor that was applied to control the Ph-negative portion of the disease. As visible in Figure 4B, synergistic effects on cell viability were obtained when combining bosutinib with HU. The other drug combinations were also effective, but due to the overwhelming effect of ponatinib, no clear synergistic effect was demonstrable (Figure 4B).

## DISCUSSION

Despite the availability of novel BCR-ABL1 TKI and SCT, advanced CML remains a challenge in clinical hematology. The prognosis of patients who develop blast crisis during TKI therapy is particularly poor. We here report on an elderly non-transplantable CML patient who developed blast crisis after having received several different TKI for over 6 years. In this particular patient, we were able to induce a complete molecular response with ponatinib. However, because of overt side effects with severe life-threatening thrombocytopenia and-later-the risk of fatal thrombosis, we had to adjust the overall treatment plan following the principles of personalized medicine, where drugs are selected or even combined to optimize efficacy and minimize (the risk of) side effects at the same time [24]. In our case, we applied ponatinib and bosutinib in rotation-cycles, thereby avoiding ponatinib-induced side effects and maintained MR4.5 at the same time.

Although BCR-ABL1 is a major driver and player in the pathogenesis of CML, multiple additional lesions and pathways are considered to contribute to disease progression and drug resistance in advanced disease [2, 25, 26]. Such additional pathways and molecules supposedly lead to a complex clonal architecture, with multiple sub-clones and diverse features that may be quite different from that of the original dominant clone. Sometimes, even, progression to an acute Ph-negative leukemia is seen [27]. In our patient, disease progression was associated with clonal evolution of Ph-positive sub-clones, as evidenced by the demonstration of additional *BCR-ABL1* mutations, but also with occurrence of a Ph-negative sub-clone that presented as an overt MDS/MPN-like disease with massive BM fibrosis. The clonal *HUMARA* pattern did not change during the time of evolution of this Ph-negative sub-clone, suggesting that both the Ph-positive and Ph-negative portion of the disease were probably indeed monoclonal. However, unfortunately, we were not able to perform deep sequencing studies to confirm our assumption.

As mentioned above, TKI resistance remains a challenge in the treatment of CML, especially when the patient is resistant to all available TKI. In our patient, the CML clone was resistant against imatinib, dasatinib, and nilotinib, and at this time, neither ponatinib nor bosutinib was available. Therefore, we had to maintain the patient on nilotinib with the hope that at least some of the relevant sub-clones can be suppressed and other novel TKI will be available. Indeed, after several months, we were able to offer ponatinib. Based on the recommendations of the European Leukemia Net and local guidelines, a switch from one to another second- or third generation BCR-ABL1 TKI can be regarded as standard. However, the application of continuous rotation-cycles using two of these TKI in advanced CML must be regarded as a novel approach. More recently, TKI rotation has also been described as a safe and effective approach in freshly diagnosed patients with CML [28].

Based on the overall situation in our case, we had to treat the patient with these rotation cycles because of the biology of the disease (only ponatinib was able to control the *BCR-ABL1*+ portion of the leukemia), because of toxicity issues, and especially because of the risk of development of thromboembolic events during ponatinib therapy [21, 22]. In addition, we tried to minimize the risk of thrombosis by co-administering aspirin and by keeping the patient on a relatively low dose of ponatinib (30 mg/day). Moreover, the patient received HU in order to keep the Ph-negative sub-clone under control. This goal was indeed achieved. However, HU may also have a protective effect against thromboembolic events and may also exert some effects on *BCR-ABL1*-mutated sub-clones. In addition, our data suggest that HU exerts strong synergistic anti-leukemic effects on CML cells when combined with bosutinib or ponatinib (M.S. and P.V., unpublished observation).

Personalized medicine has a stringent definition and is based on the assumption, that optimal drugs can be selected for distinct subgroups of patients, based on the known side effect profiles and efficacy profiles as well as knowledge about risk factors (for the development of side effects) and related patient-specific variables [24]. In most cancer types, personalized medicine is currently still under development. In CML, however, we have already the opportunity to apply drugs and to select patients in a personalized medicine-based manner. Indeed, the patient described here is a good example how a personalized medicine concept can be applied. The application of such a personalized regimen allowed us to manage a severe relapsing disease as well as to avoid potential (and to overcome obvious) side effects so that treatment could be continued with optimal response and satisfactory quality of life.

Severe thrombocytopenia is a well-known side effect of ponatinib [20, 21]. In our patient, thrombocytopenia was managed effectively by the rotation-approach. A second potentially life-threatening side effect of ponatinib is thromboembolism, which may occur preferentially in elderly, comorbid, patients [21]. We tried to avoid this side-effect by TKI-rotation therapy and co-administered aspirin. Third, the patient was suffering from severe constipation which is most probably attributable to the KIT-targeting effects of TKI on the pacemaker cells of the GI tract (cells of Cajal are KIT-dependent). The constipation resolved completely after switching from ponatinib to bosutinib, a drug that spares KIT. Finally, the recurrent pleural effusions that had developed under dasatinib and were recurrent during initial treatment with bosutinib, almost resolved when the patient switched to rotation-TKI therapy and co-administered prednisolone. All in all, the obvious advantages of the rotation have led to a long-lasting symptom-free period of CMR and a normal quality of life in our patient, which may be regarded as a triumph of personalized medicine. Interestingly, TKI rotation has recently also been applied in patients with freshly diagnosed CML using nilotinib and imatinib, with encouraging results and a reduced rate of vascular and metabolic events [28]. However, rotation therapy with ponatinib and bosutinib, although following the same principle, must be regarded as experimental approach. In fact, based on our case observation, clinical studies are now warranted in order to define whether TKI rotation is indeed a safe and effective strategy in patients with advanced TKI-refractory CML.

*In vitro* drug testing is a powerful approach to confirm or predict clinical responses, especially when patient-derived cells or CML cell lines are tested [32, 29, 30]. In the current study, we were able to show that the patient-derived leukemic blasts were responsive against HU, ponatinib, and bosutinib. In addition, all three drugs were found to counteract growth in two human CML cell lines, KU812 and K562. Moreover, we were able to show that ponatinib and bosutinib produce clear synergistic effects on growth and apoptosis of KU812 cells and some cooperative anti-leukemic effects in K562 cells. In addition, we were able to show that sequential application of ponatinib and bosutinib *in vitro*, which was performed to mimic exposure conditions *in vivo*, results in major cooperative drug effects in KU812 cells. Finally, we were able to show in a high-capacity screen that the patient-derived leukemic blasts are responsive to bosutinib and ponatinib, and that these TKI synergize with HU in blocking the viability of her cells. These data strongly suggest that drug responses of primary cells (and drug combination effects) may be predicted by drug testing and that synergistic anti-CML effects can be achieved by combining second generation TKI with each other or with HU. In fact, the patient also received HU together (in combination) with TKI. Therefore, some of the (unexpectedly strong) effect of therapy may be due to this TKI+HU combination. Based on the sub-clone concept of stem cell evolution in CML [31, 32] the clinical effect is best explained by TKI-induced eradication of stem cell-derived sub-clones [33] which was confirmed by demonstrating the complete disappearance of *BCR-ABL1*-bearing cells in our patient.

In conclusion, we present a patient with TKI-resistant multi-mutated blast crisis and a Ph-negative sub-clone producing severe BM fibrosis, in whom we induced a continuous CMR with TKI-rotation therapy complemented by HU, without major side effects. Such treatment strategies are in line with personalized medicine [24] and recent developments in the field [28] and may lead to the design of new improved treatment concepts in TKI-resistant CML. Eventually, such novel treatment concepts may also assist in the early eradication of all relevant CML sub-clones at diagnosis, so that it may be possible to switch back to a less-toxic TKI at CMR, and later discontinue TKI therapy in most patients with (even advanced) CML in the future.

### Ethics approval and consent to participate

All studies, including kinase blocker studies (IRB number 224/2006), bone marrow investigations (IRB number 1184/2014), and *in vitro* multi-drug testing (IRB number 1830/2015), were approved by the ethics committee of the Medical University of Vienna. The patient provided written informed consent to participate in these studies.

### Consent for publication

The patient gave written informed consent that all her results from clinical and laboratory investigations can be submitted and published in anonymized form in a peer-reviewed journal.

### Competing interests

PV received honoraria from Novartis, Pfizer, BMS, and Ariad, and research grants from Novartis and Ariad. The authors declare no other competing interests.

## MATERIALS AND METHODS

### Case report

A 78-year-old female patient with multi-resistant Ph+ CML was referred because of blast crisis in July 2013. She had first been diagnosed with CML in 2001 in another hospital. Initially, she had received interferon-alpha and cytarabine, and later, from May 2002, imatinib at 400 mg daily. However, after an initial response, resistance against imatinib developed, and she was referred to our center in 2005. At that time accelerated CML was diagnosed and she received a combination of rapamycin and hydroxyurea (HU). However, despite cytoreduction, no major molecular response was obtained. Subsequently, she received dasatinib (2 × 70 mg/day) and entered a complete cytogenetic response (CCyR). However, in 2007, she developed pleural effusion, and after the dose of dasatinib was reduced to 100 mg/day, a dasatinib-resistant sub-clone bearing the *BCR-ABL1* mutation F317L emerged. Treatment with nilotinib (2 × 400 mg/day) was introduced. In response to nilotinib, the patient entered a second CCyR, and *BCR-ABL1* mRNA levels decreased to < 0.1% (of *ABL1*) in September 2008. However, in December 2009, *BCR-ABL1* mRNA levels increased to 0.5%. Subsequently, the *BCR-ABL1* mutation L248V was detected. During the next few months, *BCR-ABL1* slowly increased further. However, because of stable blood counts and lack of alternative therapy options, she was maintained on nilotinib. In January 2011, a BM investigation revealed marked fibrosis without an increase in blast cells. Later in 2011, the patient developed transfusion-dependent anemia and thrombocytopenia (Table 1). In addition, *BCR-ABL1* increased to 30%, and both L248V and a new *BCR-ABL1* mutation, K274del, were detected. In June 2012, the patient developed an overt blast crisis. Treatment with ponatinib on a compassionate use program was initiated.

### Bone marrow studies

BM studies were performed in certain time intervals, namely every 6–12 months before the start of ponatinib, and 18 months after the start of ponatinib. Histologic examinations included routine histology parameters, a Gomori silver stain (to detect and quantify fibrosis) and immunohistochemistry using antibodies against CD34, CD117 (KIT), and CD61. Histologic and immunohistochemical examinations were performed by the indirect immunoperoxidase staining technique following generally accepted standards [34, 35]. Wright-Giemsa stained BM smears were examined for the percentage of blast cells, promyelocytes, basophils, and other BM cells as well as cellular dysplasia (atypia). In addition, molecular and cytogenetic studies were performed on aspirated BM cells.

### Molecular studies and karyotyping

*BCR-ABL1* transcripts were quantified by real-time PCR according to a published protocol using the Ipsogen *BCR-ABL*-Mbcr Kit (Qiagen, Hilden, Germany) and the LightCycler 2.0-System (Roche, Mannheim, Germany) [36]. *BCR-ABL1* mRNA levels were expressed as percent of *ABL1* mRNA after adjusting PCR-data according to the international scale (IS) [37, 38]. *BCR-ABL1* mutations were tested by Sanger sequencing of the *ABL1* TK domain (codon 207–414) after specific amplification of *BCR-ABL1* as reported [39]. Conventional cytogenetics and fluorescence *in situ*-hybridization (FISH) were performed according to published protocols [40].

### Human androgen receptor (*HUMARA*) polymorphism analysis

*HUMARA* analysis was performed using peripheral blood mononuclear cells (MNC) obtained at various time points before and during TKI therapy, following published techniques [41]. In brief, DNA was isolated from MNC using the MagNA Pure LC DNA isolation system (Roche) according to the manufacturer's recommendations. The X-chromosome inactivation (XCI) pattern was determined by PCR analysis of the polymorphic CAG-repeat region in the *HUMARA* gene which is located next to an HpaII restriction site. Genomic DNA (~250 ng) was digested with HpaII for 19 hours at 37°C. Then, aliquots of undigested and HpaII-digested DNA were subjected to PCR using published primer sequences [41]. Amplicons were analyzed on an Applied Biosystems (ABI) 3130xl genetic analyser (Foster City, CA, USA) using the Gene Scan 3.7 (ABI) software. For quantification, the areas under the peaks of the two alleles from the undigested and digested DNA were determined and the ratio of the peak area of both alleles (X1, X2) was calculated (in summary 100%). The degree of X-chromosome inactivation is presented as percent of the smaller peak (X2) in relation to the larger peak (X1). Undigested female DNA usually shows 2 peaks of similar height (in 90% of females).

### Isolation of primary cells and *in vitro* drug testing

Based on the intriguing effects of therapy with ponatinib and bosutinib on leukemic cells in this patient, we performed *in vitro* assays in order to explore whether these drugs exert synergistic growth-inhibitory effects on CML cells. In these experiments, primary patient-derived cells and the *BCR-ABL1+* cell lines KU812 and K562 were employed. KU812 cells were kindly provided by Dr.K.Kishi (Niigata University, Niigata, Japan) and K562 cells kindly provided by Dr.M.W.Deininger (University of Utah, Salt Lake City, UT, USA). Cells were cultured in RPMI 1640 medium with 10% FCS and exposed to various concentrations of ponatinib and bosutinib (both from ChemieTek, Indianapolis, IN, USA) alone or in combination (in quadruplicates). In case of drug combinations, a fixed ratio of compounds was applied essentially as described [42, 43]. After 24 or 48 hours, ^3^H-thymidine uptake and apoptosis (Annexin V-staining) were measured as reported previously [42, 43]. In a separate set of experiment, mononuclear BM cells obtained from the patient at the time of BC were cultured in RPMI 1640 medium and 10% FCS in the absence or presence of various concentrations of ponatinib, bosutinib, or hydroxyurea (Sigma, St. Louis, MO, USA). After 48 hours, uptake of ^3^H-thymidine was measured. In a separate set of experiments, KU812 cells were incubated in medium containing ponatinib (0.1, 0.5, or 1.0 nM) for 4 hours (37°C), washed, and then cultured in various concentrations of bosutinib (10–22 nM) for another 48 hours before ^3^H-thymidine was measured.

### Measurement of apoptosis in KU812 cells

KU812 cells were incubated in control medium, ponatinib (0.2 nM), bosutinib (10 nM), or a combination of both drugs at 37°C for 48 hours. Then, apoptotic cells were quantified by Annexin-V/PI staining and flow cytometry. In brief, cells were washed, resuspended in Annexin-V binding buffer for 15 minutes. Cells were then washed again, and propidium iodide (1 mg/mL) was added. After washing, cells were analyzed by flow cytometry on a FACSCalibur (Becton Dickinson, San Jose, CA, USA) to determine the percentage of Annexin-V/PI+ (apoptotic) cells.

### High capacity drug screening assay in primary CML cells

To compare drug effects on a larger scale and in the context of a broad range of available drugs, primary cells obtained from the patient were subjected to a high throughput drug testing assay essentially as reported [44]. The drug screen included all available BCR-ABL1 TKI as well as a larger number of other anti-leukemic drugs (total number of drugs applied: *n* = 200). In addition, drug combination experiments employing the combinations bosutinib + HU, ponatinib + HU, and ponatinib+bosutinib, were performed using this automated (robot-based) multi-drug screen. All studies were approved by the ethics committee of the Medical University of Vienna, Austria.

### Statistical analysis

To determine the significance-levels in differences seen between drug-exposed and untreated cells, the Student´s *t*-test was applied. Results were considered statistically significant when *p* was < 0.05. Drug combination effects (additive vs synergistic) were determined by calculating combination index (CI) values using Calcusyn software (Calcusyn, Biosoft, Ferguson, MO) as described [42, 43, 45]. A CI value >1 indicates an additive effect, whereas CI values below 1 indicate synergistic drug effects [45].

## Abbreviations

BM: Bone marrow
PB: peripheral blood
CML: Chronic myeloid leukemia
CP: Chronic phase
CCyR: Complete cytogenetic response
CMR: Complete molecular response
HU: Hydroxyurea
HUMARA: Human androgen receptor assay
IS: International scale
MR4.5: Molecular response 4.5 log below IRIS
Ph+: Philadelphia chromosome-positive
SCT: Stem cell transplantation
TK: Tyrosine kinase
TKI: Tyrosine kinase inhibitor(s)
XCI: X-chromosome inactivation.
